# Deciphering the Pyroptosis-Related Prognostic Signature and Immune Cell Infiltration Characteristics of Colon Cancer

**DOI:** 10.3389/fgene.2021.755384

**Published:** 2021-10-12

**Authors:** Ran Wei, Shuofeng Li, Guanhua Yu, Xu Guan, Hengchang Liu, Jichuan Quan, Zheng Jiang, Xishan Wang

**Affiliations:** Department of Colorectal Cancer Surgery, National Cancer Center/National Clinical Research Center for Cancer/Cancer Hospital, Chinese Academy of Medical Sciences and Peking Union Medical College, Beijing, China

**Keywords:** pyroptosis, colon cancer, tumor microenvironment, prognosis, machine learning

## Abstract

**Background:** Colon cancer (CC) remains one of the most common malignancies with a poor prognosis. Pyroptosis, referred to as cellular inflammatory necrosis, is thought to influence tumor development. However, the potential effects of pyroptosis-related regulators (PRRs) on the CC immune microenvironment remain unknown.

**Methods:** In this study, 27 PRRs reported in the previous study were used to cluster the 1,334 CC samples into three pyroptosis-related molecular patterns. Through subtype pattern differential analysis and structure network mining using Weighted Gene Co-expression Network Analysis (WGCNA), 854 signature genes associated with the PRRs were discovered. Further LASSO-penalized Cox regression of these genes established an eight-gene assessment model for predicting prognosis.

**Results:** The CC patients were subtyped based on three distinct pyroptosis-related molecular patterns. These pyroptosis-related patterns were correlated with different clinical outcomes and immune cell infiltration characteristics in the tumor microenvironment. The pyroptosis-related eight-signature model was established and used to assess the prognosis of CC patients with medium-to-high accuracy by employing the risk scores, which was named “PRM-scores.” Greater inflammatory cell infiltration was observed in tumors with low PRM-scores, indicating a potential benefit of immunotherapy in these patients.

**Conclusions:** This study suggests that PRRs have a significant effect on the tumor immune microenvironment and tumor development. Evaluating the pyroptosis-related patterns and related models will promote our understanding of immune cell infiltration characteristics in the tumor microenvironment and provide a theoretical basis for future research targeting pyroptosis in cancer.

## Introduction

Colon cancer (CC) is one of the most common malignancies of the digestive system, and it still has a high mortality (Sanoff et al., 2007). Worryingly, the recurrence and mortality rates of CC are in fact increasing (Bray et al., 2018). In spite of recent developments in treatment, the 5-years survival rate has not been significantly improved. Consequently, it is urgent to find gene signatures or biomarkers to identify the inherent genetic and epigenetic heterogeneity of CC and establish prognostic models for guiding therapy.

Numerous studies have shown that cancer cells can undergo cell death through pyroptosis, but the function of pyroptosis in tumor development and the tumor immune microenvironment are still controversial (Miao et al., 2011; Broz et al., 2020; Petley et al., 2021). Pyroptosis refers to a distinct form of programmed cell death, which is characterized by cells swelling with large ballooning bubbles emerging from the plasma membrane and releasing inflammatory cellular contents (Zhang et al., 2018; Frank and Vince, 2019). Unlike apoptosis, pyroptosis contributes to the activation of a variety of cytokines and danger-associated signaling molecules, which is accompanied with immune cell infiltration and inflammatory responses (Frank and Vince, 2019). During the process of pyroptosis, mature caspase-1 promotes the production of pro-inflammatory cytokines of the classical pathway, such as IL-1β7 and IL-18, which can recruit inflammatory cells and influence the tumor microenvironment (TME) (Dupaul-Chicoine et al., 2010; Kolb et al., 2014). Additionally, caspase-3 can be activated by antitumor drugs and promote the cleavage of gasdermin E (GSDME) into GSDME-N to switch the cell death mode from apoptosis to pyroptosis (Kayagaki et al., 2015; Tang et al., 2020). Pyroptosis can promote a tumor-suppressive environment by recruiting inflammatory cells and causing local inflammation, but it can also inhibit antitumor immunity and promote tumor development in many cancer types (Martinon et al., 2002; He et al., 2015; Van Gorp and Lamkanfi, 2019). For instance, it was reported that pyroptosis in a small fraction of cancer cells in the central hypoxic region of the tumor induces chronic tumor necrosis, which in turn inhibits antitumor immunity (Kayagaki et al., 2011). Accordingly, the role of pyroptosis in the development of CC still requires further study.

Recent studies have suggested that pyroptosis-related (PR) regulators would play a significant role in regulating pyroptosis (Knodler et al., 2014; Viganò et al., 2015; Yang et al., 2018). Gasdermin D (GSDMD) has been proved to be a direct substrate of inflammatory caspases and plays the role of the major executor of pyroptosis in macrophages (Wang et al., 2020). Studies have also proposed that GSDMD may be positively correlated with the migration and invasion of lung cancer (Zanoni et al., 2016). However, downregulation of GSDMD expression was found to promote S/G2 cell cycle transition, which indicated that GSDMD may serve as a tumor suppressor in gastrointestinal cancers (Zanoni et al., 2016). Furthermore, GSDMA/B/C was proved to be the substrate of caspases or granzymes, and the oligomerization of its N-terminal in the membrane was found to increase pyroptosis (Lee et al., 2018). In most previous studies, the function of these PR regulators was identified individually through classical approaches. However, the composition of the TME is complex, and many tumor regulators can interact in a highly coordinated manner. Therefore, comprehensively estimating the immune cell infiltration characteristics of the TME with multiple PR regulators would increase our understanding of tumor immunity and the antitumor inflammatory response.

In the current study, we established a molecular subtype classification pattern by integrating the genomic information of 1,023 CC samples based on 27 PR regulators. The CC samples were classified into three distinct PR patterns, which were associated with the tumor immune microenvironment and prognosis. Additionally, we developed a risk assessment tool related to PR regulators and defined the PR risk assessment model (PRM) scores using LASSO regression analysis and machine learning, which could be used to assess the prognosis, immune infiltration, and potential treatment targets of CC.

## Materials and Methods

### Colon Cancer Dataset Source

The workflow chart is shown in the Supplementary Data (Supplementary Figure S1). The public gene-expression data for transcriptome profiling and the corresponding clinical annotation were obtained from Gene Expression Omnibus (GEO) and The Cancer Genome Atlas (TCGA) database on May 1, 2021. There were four eligible CC cohorts of gene-expression data (GSE39582, GSE33113, and TCGA–Colon Adenocarcinoma [TCGA-COAD) (discovery data) and GSE17538 (independent validation data)]. We downloaded the raw microarray data form the Affymetrix Human Genome U133 Plus 2.0 Array of GEO database and the RNA sequencing data (fragments per kilobase of transcript million mapped reads (FPKM) value) of TCGA. We employed the “ComBat” algorithm in “SVA” package to adjust the batch effects from nonbiological technical biases among different CC RNA-seq data. And all of the RNA-seq data were adjusted for background adjustment and quantile normalization with robust multiarray averaging method in “affy” and “simpleaffy” packages. And the DNA sequencing of annotated somatic mutation of single-nucleotide polymorphisms (SNPs) and copy number variation (CNV) data for CC were also downloaded from TCGA. All CC samples were coded according to the third Edition of International Classification of Diseases for Oncology (ICD-O-3). And the exclusion criteria included patients with incomplete survival information and missing data on neoplasm histologic type.

### Identification of Pyroptosis-Related Regulators

From previous research, we identified a total of 27 PR genes presented in the Supplementary Data (Supplementary Table S1). All of PR genes were gathered from previous study and MSigDB database (Latz et al., 2013; Shi et al., 2015; Orning et al., 2018; Karki and Kanneganti, 2019; Li et al., 2021). For example, the previous study suggested that the caspase (CASP) family (CASP1, CASP3, CASP4, CASP5, CASP6, CASP8, and CASP9) was related to GSDMD, GSDMB, GSDMA, and GSDMC, which were significant for cancer cell pyroptosis (Shi et al., 2015; Li et al., 2021). And study showed that CASP3 and Granzyme B (GZMB) could help to convert cell apoptosis into pyroptosis (Orning et al., 2018). A protein–protein interaction (PPI) network for the differentially expressed genes (DEGs) was constructed with Search Tool for the Retrieval of Interacting Genes (STRING), version 11.0 (https://string-db.org/).

### Unsupervised Clustering for Colon Cancer Molecular Subtypes

We built a novel PR molecular subtype based on the level of 27 PR genes identified from three CC cohorts. The unsupervised clustering analysis clustering algorithm was performed to estimate the patterns of pyroptosis regulation and classify the CC samples for further analysis. The stability and patterns of molecular clusters were adjusted by the consensus clustering algorithm (Wong, 1979). The “ConsensuClusterPlus” package was employed to cluster, and the process was performed 1,000 times (Wilkerson and Hayes, 2010).

### Identification of Differentially Expressed Genes Among Subtypes

To identify PR regulators genes, we need to estimate the expression level of different genes for studying the molecular feature among PR subtypes. We identified the DEGs with the empirical Bayesian approach in “limma” package, and we set the |log2-fold change| > 1 and false discovery rate (FDR) < 0.05 as the significance criteria.

### Gene Set Variation Analysis and Gene Set Enrichment Analysis

To investigate the molecular feature among PR subtypes, we established gene set variation analysis (GSVA) enrichment analysis with “GSVA” R packages (Hänzelmann et al., 2013). The gene set of “c2. cp.kegg.v6.2. symbols” and “c5. all.v6.2. symbols.gmt” were gathered from the MSigDB database to be used in GSVA. H: Hallmark gene sets; C2: curated gene sets [including Kyoto Encyclopedia of Genes and Genomes (KEGG)] were downloaded from the MSigDB database to be used in gene set enrichment analysis (GSEA) with the software gsea 3.0. And we set the adjusted *p* < 0.05, nominal (NOM) *p* < 0.05, and FDR q < 0.05 as the statistically significance to identify the difference on biological process.

### Estimation of Infiltrating Immune Cells and Immune Microenvironment Characteristics

The Estimation of STromal and Immune cells in MAlignant Tumor tissues using Expression data (ESTIMATE) was used to calculate the stromal score, immune-score, tumor-purity, and ESTIMATE-score for CC (Song et al., 2017). The enrichment levels of the 29 immune signatures were established based on the genes set from MSigDB database (Supplementary Table S1) with the single-sample GSEA (ssGSEA) (Ritchie et al., 2015; Bu et al., 2021). And the infection of 22 human immune cells in TME was established with cell-type identification by estimating relative subsets of RNA transcripts (CIBERSORT) web portal (https://cibersortx.stanford.edu/) and 1,000 permutations (Chai et al., 2019). The deconvolution algorithm output had a *p*-value <0.05 was set as successful and accurate deconvolution, which would be normalized to make their direct interpretation as cell fractions for comparison across different groups.

### Weighted Gene Co-Expression Network Analysis

The Weighted Gene Co-expression Network Analysis (“WGCNA”) R package was employed to build the co-expression network of DEGs (van Houwelingen et al., 2006). The co-expression similarity matrix, Pearson’s correlation matrices, and average linkage method were involved in evaluating the correlations among the included genes. The Amn = |Cmn|β (Amn is theadjacency between gene m and gene n; Cmn, Pearson’s correlation between gene-m and gene-n; and β, soft thresholding parameter) could show that the strength of correlations contributes to the weighted adjacency matrix with a scale-free co-expression network. The topological overlap matrix (TOM) was used to identify the connectivity and dissimilarity of the co-expression network established with an appropriate β value.

### Statistical Analysis

The log-rank test and the Kaplan–Meier survival analysis were used to evaluate the difference in overall survival (OS) among different groups. We used the package “caret” to allocate all the CC patients in inner-training and inner-testing groups randomly through the 8:2 ratio, which contributed to enhance the generalization ability of model. The LASSO-penalized Cox regression model was used to evaluate the role of genes to identify signatures significantly associated with the patients’ OS. And the 10-fold cross validation was employed to prevent overfitting with the penalty parameter lambda.1se (Heagerty et al., 2000). The univariable and multivariate Cox regression analyses were used to identify the independent prognostic factors and to establish eight PR signatures and nomogram based on the forward and backward elimination methods. The area under the curve (AUC) and the time-dependent receiver operating characteristic (ROC) curve were used to evaluate the prognostic accuracy of the eight PR signatures model in inner-training and inner-testing groups with the package “survival ROC” (Pei et al., 2020). The PRM-scores were established based on the eight PR signatures model, and the median of PRM-scores was set as the cutoff value to the separate patients into high- and low-PRM-score groups. Bootstrap method was performed to validate the Cox model internally and externally. Bootstrap-corrected OS rates were calculated by averaging the Kaplan–Meier estimates based on 2,000 bootstrap samples.

## Results

### The Genetic and Expression Characteristics of Pyroptosis-Related Regulators in Colon Cancer

**FIGURE 1:**
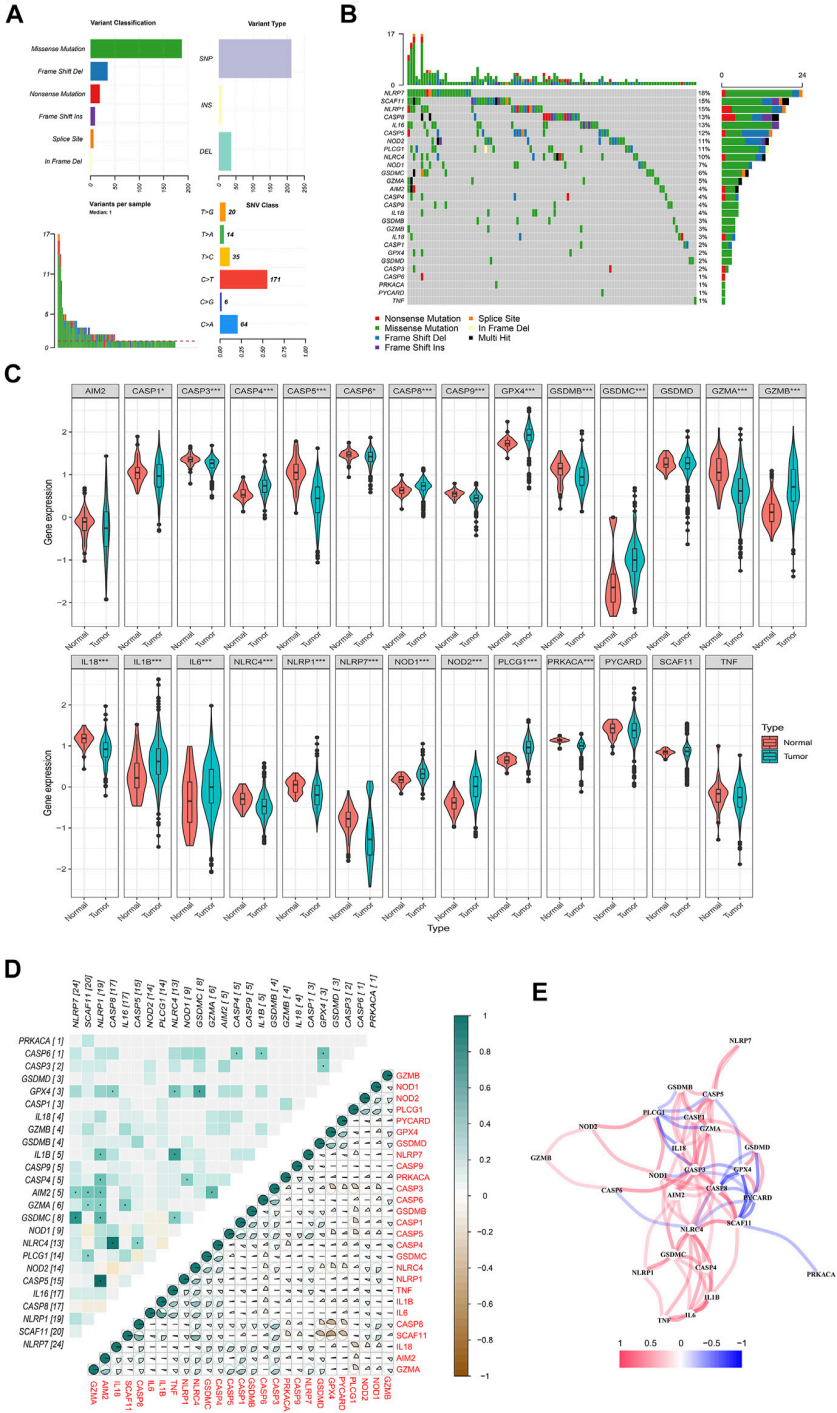
Landscape of genetic and expression variation of pyroptosis-related regulators in colon cancer. **(A, B)** The mutation frequency and classification of 27 pyroptosis-related regulators in colon cancer based on The Cancer Genome Atlas—Colon Adenocarcinoma (TCGA-COAD). **(C)** The expression of 27 pyroptosis-related regulators in colon cancer and normal tissues: tumor, blue; and normal, red. The upper and lower ends of the boxes represent the interquartile range of values. The lines in the boxes represent median value, and black dots show the outliers. The asterisks represent the statistical *p*-value. Mann–Whitney U test. *p < 0.05; **p < 0.01; ***p < 0.001; *p* ≥ 0.05, not significant. **(D)** Heatmap showing the correlation of genetic variation and expression variations of pyroptosis-related regulators. Left: genetic variation; right: expression variations. *p* < 0.05. **(E)** The correlation network of the pyroptosis-related genes (red line, positive correlation; blue line, negative correlation; the depth of the colors reflects the strength of the relevance).

A total of 27 PR regulators were identified in CC in this study with three eligible CC cohorts. We dissect the incidence of somatic mutations and molecular signatures of PR regulators in CC from TCGA-COAD (Figure 1A). The result showed that 109 of 590 CC samples experienced mutations of PR regulators, with frequency of 23.33%. It was found that the missense mutation exhibited the highest frequency variant classification. Both C>T ranked and SNPs were the most frequent alternatives in single-nucleotide variant (SNV) class and variant type. The NLRP7 exhibited the highest alteration frequency followed by SCAF11, while the PRKACA, CASP6, PYCARD, and TNF showed extremely low alteration frequency in CC samples (Figure 1B). To ascertain whether the above genetic variations influenced the expression of PR regulators in CC patients, we investigated the mRNA expression levels of regulators between normal and CC samples (Mann–Whitney U test; *p < 0.05; **p < 0.01; ***p < 0.001; *p* ≥ 0.05, not significant) (Figure 1C). The expression of CASP4, GASP8, GPX4, GSDMC, GZMB, IL1B, NOD1, NOD2, and PLCG1 was increased; while the expression of AIM2, CASP1, CASP3, CASP5, CASP6, CASP9, GSDMB, GZMB, IL18, NLRP1, and NLRP7 was decreased in CC samples compared with normal tissues. Correlation analysis was performed with genetic variation and expression variations of PR regulators in CC to further investigate the relationship among these regulators (left: genetic variation; right: expression variations) (Figure 1D). The correlation network containing all PR genes is presented in Figure 1E (red: positive correlations; blue: negative correlations).

### Construction of a Molecular Subtype Classification Pattern for Colon Cancer Mediated by 27 Pyroptosis-Related Regulators

**FIGURE 2:**
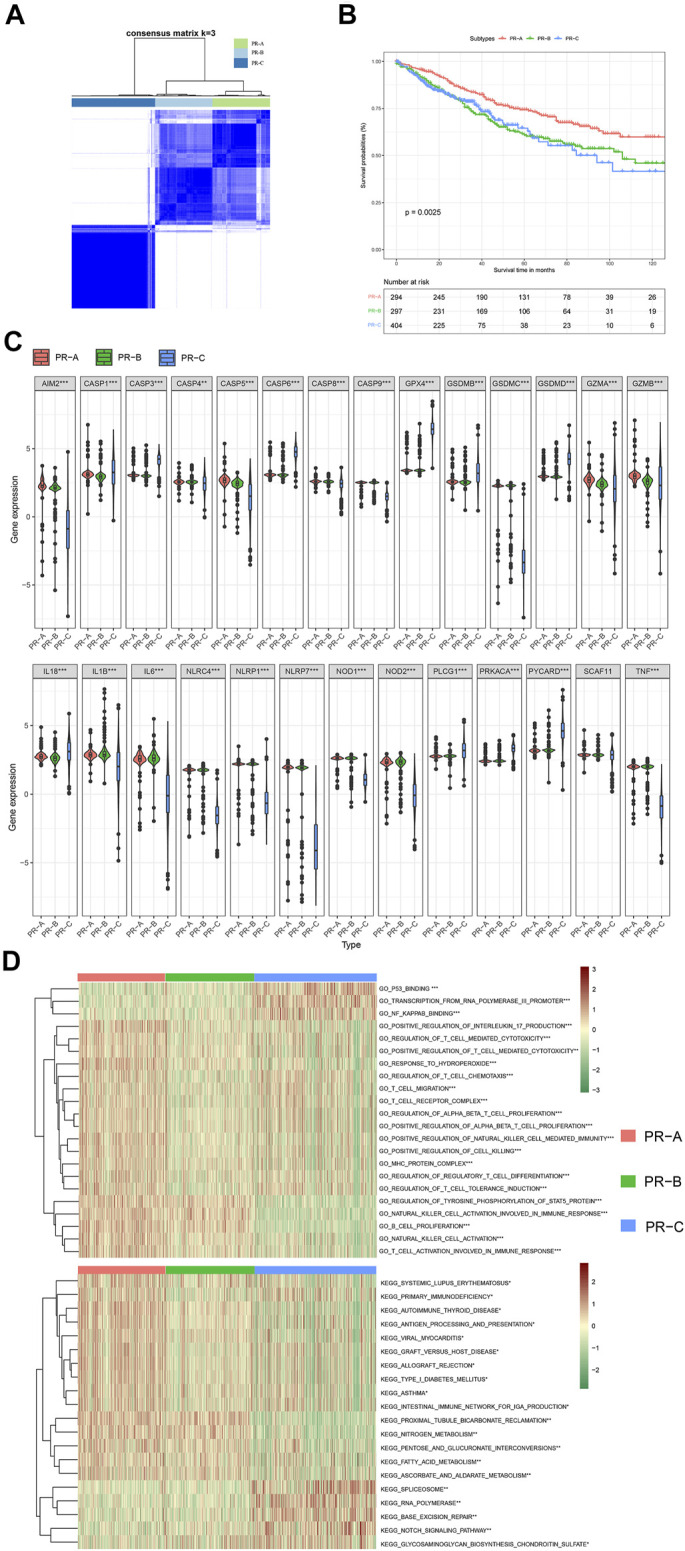
Subgroups of colon cancer related by pyroptosis-related regulators. **(A)** The consensus score matrix of all colon cancer patients when k = 3 in three cohorts based on the three eligible colon cancer (CC) cohorts of gene-expression data (GSE39582, GSE33113, and The Cancer Genome Atlas—Colon Adenocarcinoma (TCGA-COAD)). Two samples were more likely to be grouped into the same cluster when there was a higher consensus score between them in different iterations. **(B)** OS curves for the three pyroptosis-related (PR) clusters based on colon cancer patients from three cohorts (log-rank test, *p* < 0.01). OS, overall survival. **(C)** The expression of 27 pyroptosis-related regulators in three PR clusters: PR-A, red; PR-B, green; and PR-C, blue. The upper and lower ends of the boxes represent the interquartile range of values. The lines in the boxes represent median value, and black dots show outliers. The asterisks represent the statistical *p*-value. ANOVA test. *p < 0.05; **p < 0.01; ***p < 0.001; *p* ≥ 0.05, not significant. **(D)** These heatmaps were employed to visualize Gene Ontology (GO) and Kyoto Encyclopedia of Genes and Genomes (KEGG) analyzed by gene set variation analysis (GSVA), which presented the enrichment biological pathways in distinct three PR clusters (Bayes moderation, ∗*p* < 0.05; ∗∗*p* < 0.01; ∗∗∗*p* < 0.001).

To explore the potential biological molecular of PR regulators, we established a PR molecular subtype using consensus clustering analysis for CC patients. Three CC datasets with available clinical and follow-up information (GSE39582, GSE33113, and TCGA-COAD) were incorporated into one meta-cohort and clustered into three molecular subtypes (PR-A, PR-B, and PR-C) based on the expression of 27 PR regulators (Figure 2A). There are high intragroup correlations and low intergroup correlations in this classification pattern. There was also a significant difference in the survival among three subtypes (Figure 2B). The results of survival analysis proved that the OS of the PR-B and PR-C groups was significantly lower than that of the PR-A group according to the Kaplan–Meier curves of the CC cohorts (log-rank test, *p* < 0.01, Figure 2B). The expression of 27 PR regulators was different in three subtypes (ANOVA test, *p < 0.05; **p < 0.01; ***p < 0.001; *p* ≥ 0.05, not significant) (Figure 2C). In order to further portray the biological characteristics of these distinct molecular subtypes, we established GSVA enrichment analysis, including the KEGG and Gene Ontology (GO). The PR-A showed enrichment in terms of pathways associated with immune activation, including IL-17 production, T cell-mediated cytotoxicity, T cell-mediated, T-cell chemotaxis, and T-cell migration and differentiation. PR-B presented enrichment pathways including the proximal tubule bicarbonate reclamation, nitrogen metabolism, and tyrosine phosphorylation of STAT5 protein. While the enrichment pathways in PR-C were associated with immune suppression, including downregulation in natural killer (NK) cell activation involved in immune response, B-cell proliferation, and T-cell activation involved in immune response.

### Different Characteristics of Tumor Microenvironment Cell Infiltration Among Three Pyroptosis-Related Subtypes

**FIGURE 3:**
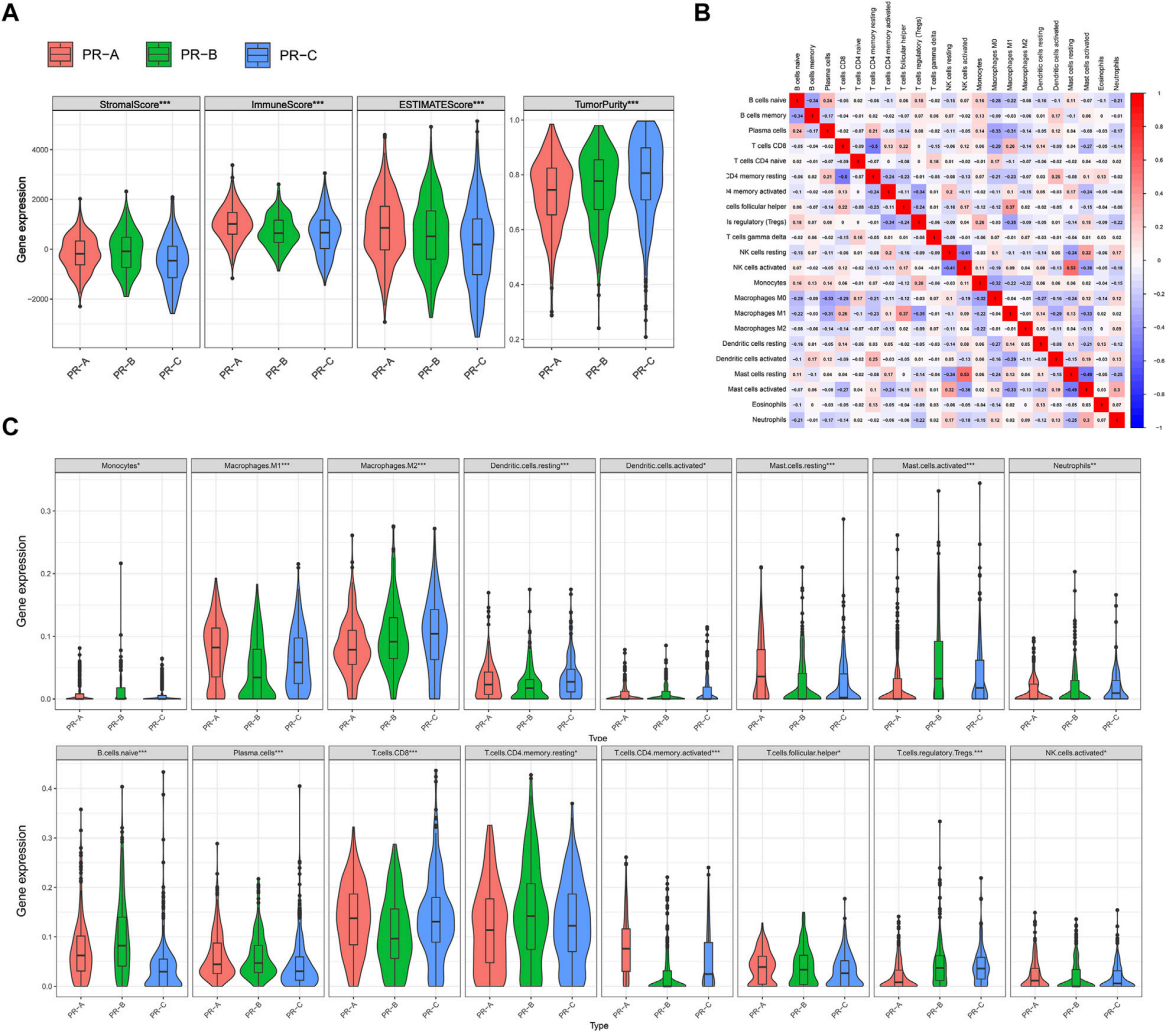
Distinct three pyroptosis-related (PR) clusters showed diverse tumor microenvironment (TME) cell infiltration. **(A)** The level of stromalScores, immuneScores, ESTIMATEScores, and tumorPurity calculated with ESTIMATE in three PR clusters based on the three eligible colon cancer (CC) cohorts of gene-expression data (GSE39582, GSE33113, and The Cancer Genome Atlas—Colon Adenocarcinoma (TCGA-COAD)). **(B)** Heatmap showing the correlation of TME cell infiltration calculated with CIBERSORT. **(C)** The level of TME cell infiltration in three PR clusters: PR-A, red; PR-B, green; and PR-C, blue. The upper and lower ends of the boxes represent the interquartile range of values. The lines in the boxes represent median value, and black dots show outliers. The asterisks represent the statistical *p*-value. ANOVA test. *p < 0.05; **p < 0.01; ***p < 0.001; *p* ≥ 0.05, not significant.

In addition, we tend to estimate the immune microenvironment among the PR molecular subtypes. The TME cell infiltration characteristics were calculated with ESTIMATE, including the tumor purity and immune-scores (ANOVA test, *p < 0.05; **p < 0.01; ***p < 0.001; *p* ≥ 0.05, not significant) (Figure 3A). The result showed that the immune-scores and ESTIMATE were the highest in PR-A among three subtypes, which suggested that the PR-A presented a high level of immune fully activation. The highest stromal-scores and tumor purity were in PR-C, and the lowest immune-scores were in PR-C, which suggested that the PR-C may characterized by the suppression of immunity. To investigate the proportions and differences of tumor infiltrating immune cell subsets among PR regulators subtypes, we employed a deconvolution algorithm with the CIBERSORT method (Figure 3B, Supplementary Figures 1A,B). The results noted that there were significant differences on the compositions of TME cell types among the three PR subtypes, which suggested that PR regulators may influence the types of TME infiltrating cell in CC. We found that the infiltration of activated immune cell in TME was abundant in PR-A, including the presence of CD8 T cells, activated NK cells, and B cells (ANOVA test, *p < 0.05; **p < 0.01; ***p < 0.001; *p* ≥ 0.05, not significant) (Figure 3C), which were same with the immune-scores from ESTIMATE. The high level of immunity may be related to the significant survival advantage (Bai et al., 2020). The PR-B was enriched with M1 macrophages, dendritic cells, plasma cells, and CD8 T cells. And the PR-C was enriched with M2 macrophages, naive B cell, CD4 T-cell memory resting, and T-cell regulatory cells (Tregs). The PR-C was reached with M2 macrophages, resting dendritic cells, and Tregs. And we quantify the enrichment levels of immunity related pathways and immune cells in CC via ssGSEA with a total of 29 immune-associated gene sets (Supplementary Figure S2). There was a significant difference in level of HLA genes among three subtypes. The checkpoint, CD8 T cells, HLA, MHC, and TILs were the highest in PR-A, which suggested the potentially ability for immune-inflamed. Based on the characterization of TME cell infiltration and biological molecular, PR-A was classified as immune-activated phenotype, with abundant immune cell infiltration and survival advantage; PR-B was classified as intermediate phenotype; and PR-C was classified as immune-excluded phenotype, characterized by the low immune response and high tumor purity. But the type of TME immune cells was the same among different subtypes, which showed that the PR regulators may regulate the level of immune cell infiltration and that they could not influence the types of cells in TME.

### Development and Validation of Risk Assessment Tool-Constructions Related to Pyroptosis-Related Regulators for Colon Cancer Patients

**FIGURE 4:**
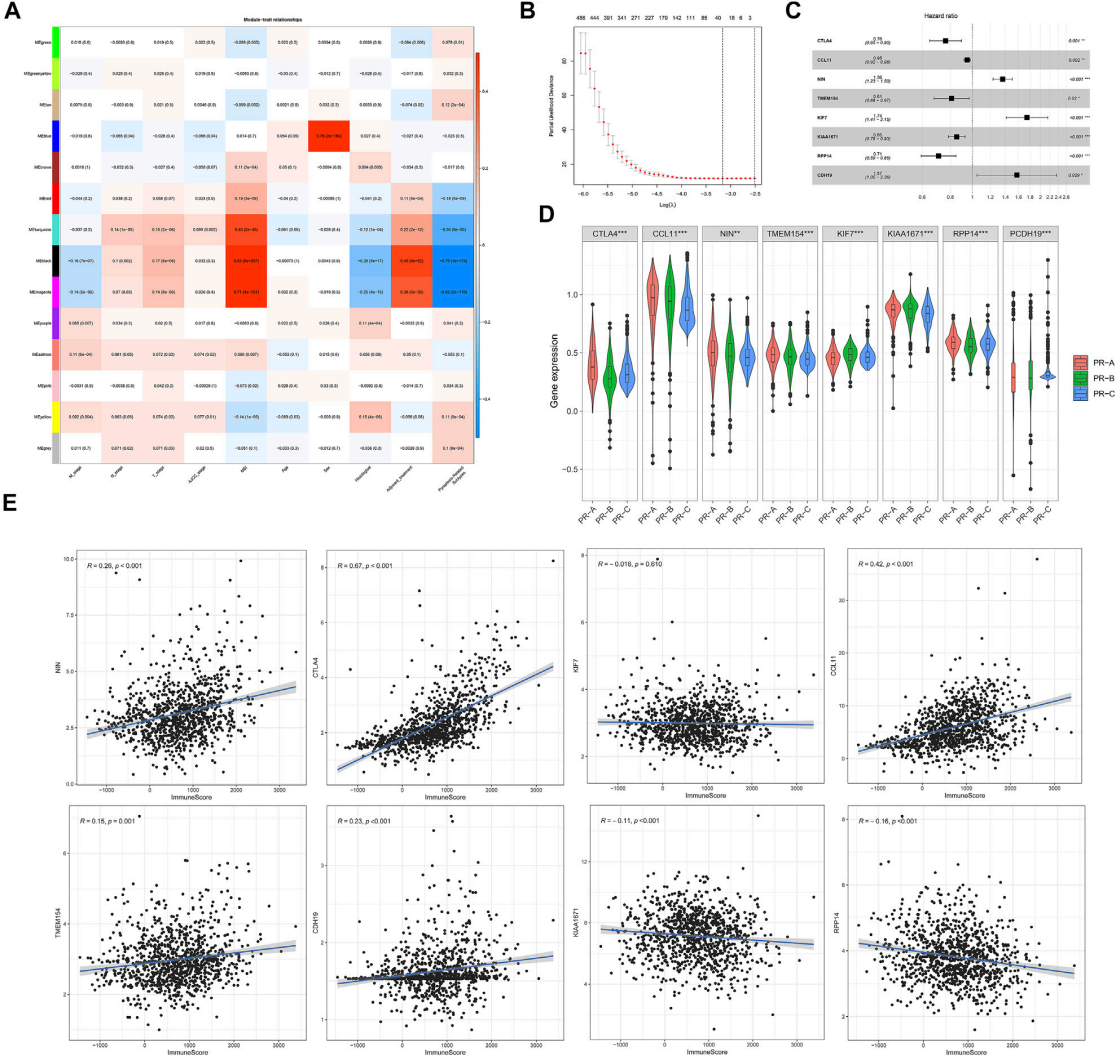
Generation of risk assessment tool-constructions to predict patient survival related to pyroptosis-related regulators for colon cancer patients. **(A)** Identification of a co-expression module in colon cancer. Each piece of the leaves on the cluster dendrogram corresponded to a gene, and those genes with similar expression patterns compose a branch. Correlation between gene modules and clinical features or three pyroptosis-related (PR) clusters. The upper row in each cell indicates the correlation coefficient ranging from −1 to 1 of the correlation between a certain gene module and clinical features or three PR clusters. The lower row in each cell indicates the *p*-value. **(B)** In the LASSO-Cox model of inner-training cohort from GSE39582, GSE33113, and The Cancer Genome Atlas—Colon Adenocarcinoma (TCGA-COAD) data, the minimum standard was adopted to obtain the value of the super parameter l by 10-fold cross validation. **(C)** Hazard ratio and *p*‐value of the constituents involved in multivariate Cox regression analyses of eight signatures in inner-training cohorts. **(D)** The expression of eight signatures in three PR clusters: PR-A, red; PR-B, green; and PR-C, blue. The upper and lower ends of the boxes represent the interquartile range of values. The lines in the boxes represent median value, and black dots show outliers. The asterisks represent the statistical *p*-value. ANOVA test. *p < 0.05; **p < 0.01; ***p < 0.001; *p* ≥ 0.05, not significant. **(E)** The association between the immuneScores calculated with ESTIMATE and the expression of eight signatures related to three PR clusters.

To further reveal the role of PR subtypes for prognosis and treatment of CC and apply the clusters to guide subsequent treatment, we established risk assessment tool-constructions based on the PR subtypes. All the genes were analyzed for co-expression network analysis using the WGCNA package (Figure 4A, Supplementary Figure S3). The association was built among the expression of gene and the PR clusters and clinical information based on the three eligible CC cohorts of gene-expression data (GSE39582, GSE33113, and TCGA-COAD). A total of 18 modules were identified; and the ME in the brown, yellow, red, and pink modules showed significantly higher association with PR regulators clusters than other modules in CC. From these modules, we identified 854 signature genes associated with the PR regulators (*p* < 0.05), which were selected for further analysis. Next, we estimated the independent prognostic signature of these genes using univariate Cox regression analysis, and the *p*-value <0.05 was considered to be the cutoff criteria. Patients from TCGA-COAD, GSE33113, and GSE39582 were randomly divided into inner-training and inner-testing groups through the 8:2 ratio. And we set GSE17538 as the independent validation cohort. Next, we established the LASSO-Cox regression model and cross validation to calculate the mean-squared error of genes with independent prognostic factors (Figure 4B). Eight genes, cytotoxic T-lymphocyte-associated protein 4 (CTLA4), chemokine (C-C motif) ligand 11 (CCL11), ninein (NIN), transmembrane protein 154 (TMEM154), kinesin family member 7 (KIF7), KIAA1671, ribonuclease P/MRP 14-kDa subunit (RPP14), and cadherin 19 (CDH19), were identified with the LASSO-Cox regression model and multivariate Cox regression analysis, which were used to establish the PRM (Figure 4B). All of these genes had significant independent prognostic factors in multivariate Cox regression analysis (Figure 4C). Besides these, eight genes expression were different in three PR subtypes (ANOVA test, *p < 0.05; **p < 0.01; ***p < 0.001; *p* ≥ 0.05, not significant) (Figure 4D). And CTLA4, CCL11, NIN, TMEM154, KIAA1671, RPP14, and CDH19 expressions were associated with immune-scores (Figure 4E). The prognostic index formula for CC was as follows: PRM-scores = [Status of *CTLA4* * (−0.27274) + Status of *CCL11* * (−0.05312) + Status of *NIN* * (0.30814) + Status of *TMEM154* * (−0.21183) + Status of *KIF7* * (0.55555) + Status of *KIAA1671* * (−0.15928) + Status of *RPP14* * (−0.34418) + Status of *CDH19* * (0.45252)]. We divided colon patients into high- and low-PRM-score groups based on the median value, which was set as the cutoff value to divide the patient into high or low group in the validation cohorts.

**FIGURE 5:**
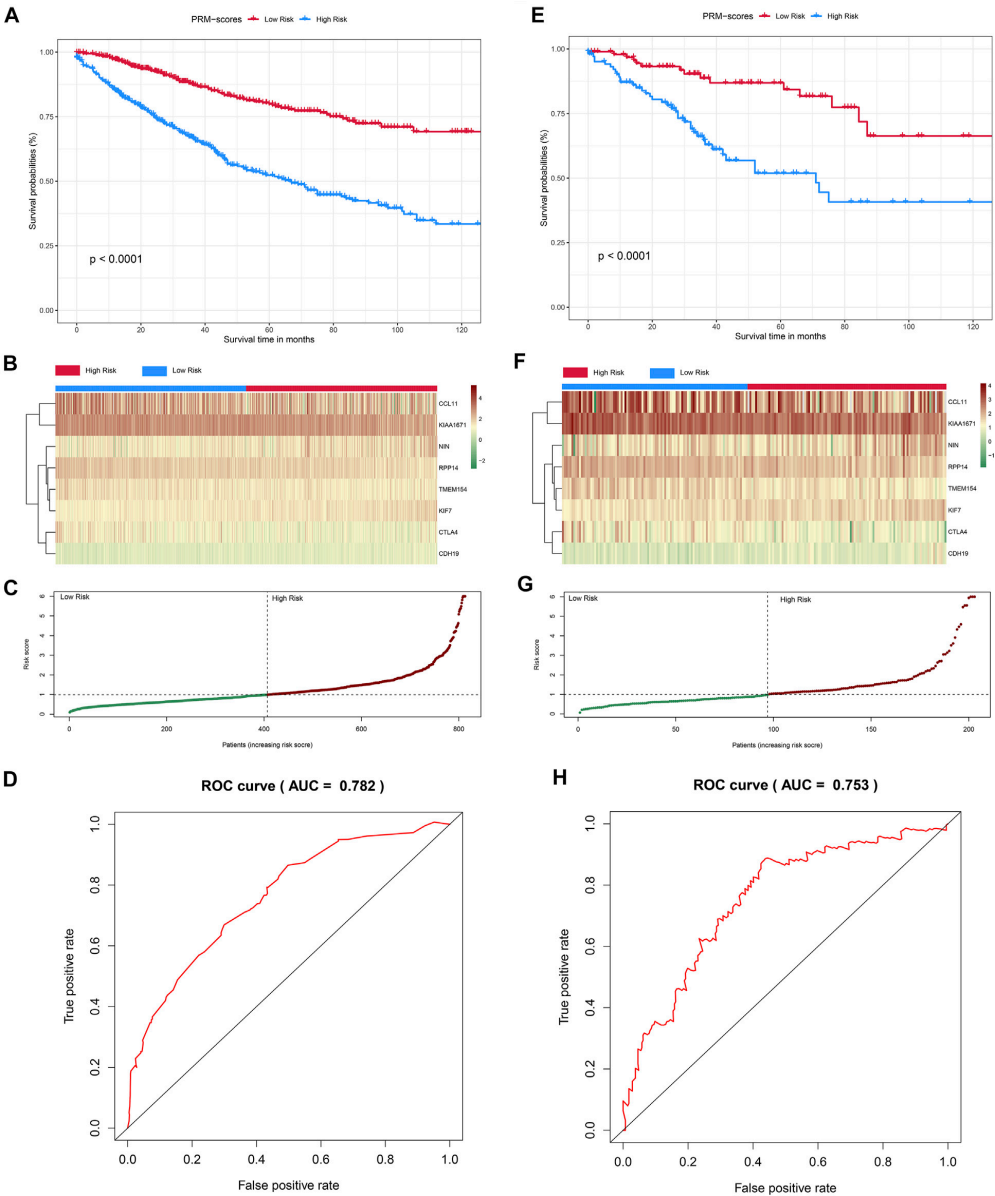
Construction and validation of the PRM-scores in colon cancer cohorts. **(A)** Kaplan–Meier curves for the overall survival (OS) of colon patients in inner-training cohort between the high- and low-PRM-scores groups based on GSE39582, GSE33113, and The Cancer Genome Atlas—Colon Adenocarcinoma (TCGA-COAD) data (log-rank test, *p* < 0.01). **(B, C)** Distribution of PRM-scores and the expression of eight signatures related to three pyroptosis-related (PR) clusters in inner-training cohort. **(D)** receiver operating characteristic (ROC) curves demonstrated the predictive efficiency of the PRM-scores in inner-training cohort. **(E)** Kaplan–Meier curves for the OS of colon patients in validation cohort between the high- and low-PRM-scores groups (log-rank test, *p* < 0.01). **(F, G)** Distribution of PRM-scores and the expression of eight signatures related to three PR clusters in validation cohort. **(H)** ROC curves demonstrated the predictive efficiency of the PRM-scores in validation cohort.

The survival analysis suggested that the OS of the high-PRM-score group was significantly lower than that of the low-PRM-score group in inner-training cohort (log-rank test, *p* < 0.001, Figure 5A), as well as the Kaplan–Meier curves of the inner-testing cohort (log-rank test, *p* < 0.001, Figure 5E). The PRM-score distribution and the expression of eight PR significant genes in the inner-training and inner-testing cohorts are presented in Figures 5B,C,F–G. Then, ROC curves were used to estimate the validity of the eight PR risk assessment tool-constructions in CC cohorts. The AUCs were equal to 0.738 at 3 years and 0.782at 5 years in the inner-training group (Figure 5D, Supplementary Figures 4A, B). Similarly, the AUCs were equal to 0.708 at 3 years and 0.753 at 5 years in the inner-testing group (Figure 5H, Supplementary Figure 4C), which showed that the model could achieve satisfactory predictive accuracy in both the inner-training and inner-testing cohorts. We established the survival analysis and ROC curves in the independent validation cohort (GSE17538), which showed the significant difference in OS between high- and low-PRM-score groups (log-rank test, *p* < 0.001, Supplementary Figure 5A). The AUCs were equal to 0.644 at 3 years and 0.684 at 5 years in the independent validation group (Supplementary Figures 5B, C). And we established the model to predict the prognosis based on PR genes with “random Survival Forest” (Supplementary Figures 6A–C). And the 10-fold cross validation was employed to prevent overfitting.

### Differences of Immune Function and Biological Characteristic Between Risk Assessment Model-Scores Groups

**FIGURE 6:**
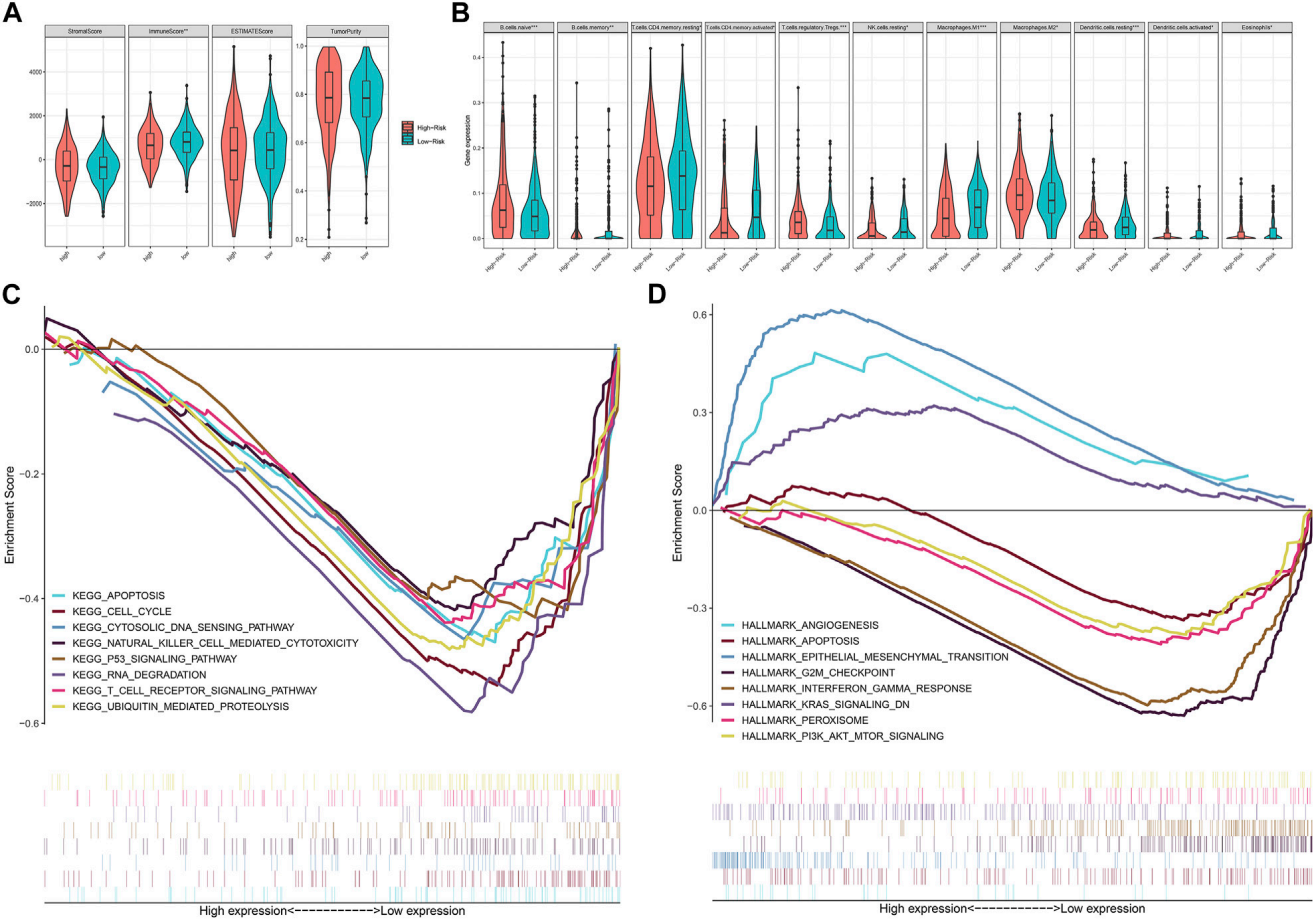
Characteristics of the PRM-scores scoring model for colon cancer patients. **(A)** The level of stromalScores, immuneScores, ESTIMATEScores, and tumorPurity calculated with ESTIMATE in high- and low-PRM-scores groups. **(B)** The level of tumor microenvironment (TME) cell infiltration in high- and low-PRM-scores groups: high PRM-scores, red; and low PRM-scores, blue. The upper and lower ends of the boxes represent the interquartile range of values. The lines in the boxes represent median value, and black dots show outliers. The asterisks represent the statistical *p*-value. Mann–Whitney U test. *p < 0.05; **p < 0.01; ***p < 0.001; *p* ≥ 0.05, not significant. **(C)** The enriched gene sets in Kyoto Encyclopedia of Genes and Genomes (KEGG) by samples with high-risk sample. And only several leading gene sets are displayed in the plot. **(D)** Enriched gene sets in Hallmark collection by samples of high-risk sample. Only several leading gene sets are shown in the plot. Each line represents one particular gene set with unique color, and upregulated genes are located in the left approaching the origin of the coordinates; by contrast, the downregulated genes are on the right of the *x*-axis. Only gene sets with nominal (NOM) *p* < 0.05 and false discovery rate (FDR) q < 0.05 were considered significant. And only several leading gene sets are displayed in the plot.

We estimated the immune microenvironment between the eight genes related high- and low-PRM-score groups. Only the immune-scores were significant different between two groups, and the level of tumor-purity was the same in the two groups (Mann–Whitney U test, *p < 0.05; **p < 0.01; ***p < 0.001; *p* ≥ 0.05, not significant) (Figure 6A). We found that the infiltration of activated immune cell in TME was abundant in low-PRM-score groups, including the M1 macrophages, NK cells, CD4 T cells (Mann–Whitney U test, *p < 0.05; **p < 0.01; ***p < 0.001; *p* ≥ 0.05, not significant) (Figure 6B). To further evaluate the association between the expression of the tumor immune microenvironment and these eight genes, we analyzed the corrections between the 22 types of immune cell infiltration profiles and these eight genes (Supplementary Figure S7). GSEA was used to analyze potential biological characteristics of the PRM-score groups in CC patients. As shown in Figures 6C,D, according to the Hallmark and KEGG collection defined by MSigDB, the genes in the high-PRM-score group were mainly enriched in angiogenesis, KRAS signaling, and epithelial mesenchymal transition. And the genes in the low-PRM-score groups were mainly enriched in cell cycle, P53 signaling pathway, T-cell receptor signaling pathway, and PI3K/AKT/MTOR signaling.

### Establishment and Validation of the Nomogram

The univariate and multivariable Cox regression models were applied to the inner-training cohort to evaluate the predictors of OS. Univariate analyses indicated that age, stage-N, stage-M, PRM-scores, and PR subtypes were associated with OS in CC patients (*p* < 0.05 in all cases, Table 1). Next, the multivariate Cox analyses found that age, stage-N, stage-M, PRM-scores, and PR subtypes were independent risk factors for OS based on forward and backward elimination methods (Table 1).

**TABLE 1:** Univariable and multivariable Cox regression analyses of OS in CC patients.

Characteristic (OS)	Univariable analysis		Multivariable analysis	
	HR (95% CI)	*p*-Value	HR (95% CI)	*p*-Value
Age (years)	—	—	—	—
<60	1	—	1	—
≥60	1.24 (1.93–1.67)	0.031	1.44 (1.07–1.94)	0.016
Gender	—	—	—	—
Female	1	—	—	—
male	1.28 (0.99–1.65)	0.056	—	—
T stage	—	—	—	—
T1/2	1	—	1	—
T3/4	1.73 (1.06–2.85)	0.029	1.24 (1.15–2.05)	0.043
Unknown	3.74 (1.71–5.18)	<0.001	0.98 (0.26–3.71)	0.981
N stage	—	—	—	—
N0	1	—	1	—
N1/2	1.78 (1.38–2.32)	<0.001	0.20 (0.90–1.64)	0.181
Unknown	2.59 (1.50–4.48)	<0.001	1.73 (1.25–4.33)	0.021
M stage	—	—	—	—
M0	1	—	1	—
M1	4.58 (3.36–6.24)	<0.001	3.55 (2.51–3.70)	<0.001
Unknown	2.69 (1.74–4.19)	<0.001	2.09 (1.17–3.74)	0.002
PRM-scores	—	—	—	—
Low	1	—	1	—
High	3.76 (2.82–5.01)	<0.001	3.17 (2.36–4.26)	<0.001
Pyroptosis-related molecular subtype	—	—	—	—
PR-A	1	—	1	—
PR-B	1.69 (1.25–2.31)	<0.001	1.31 (1.09–1.81)	0.013
PR-C	1.78 (1.28–2.49)	<0.001	1.27 (1.09–1.82)	0.034

Note. Multivariate Cox regression analysis is used to calculate the HRs and 95% CIs for OS in CC patients. Covariables that are significant in univariable competing risk regression analysis (*p* < 0.05) are included in the multivariable analysis.

HR, hazard ratio; CI, confidence interval; CC, colon cancer; OS, overall survival.

**FIGURE 7:**
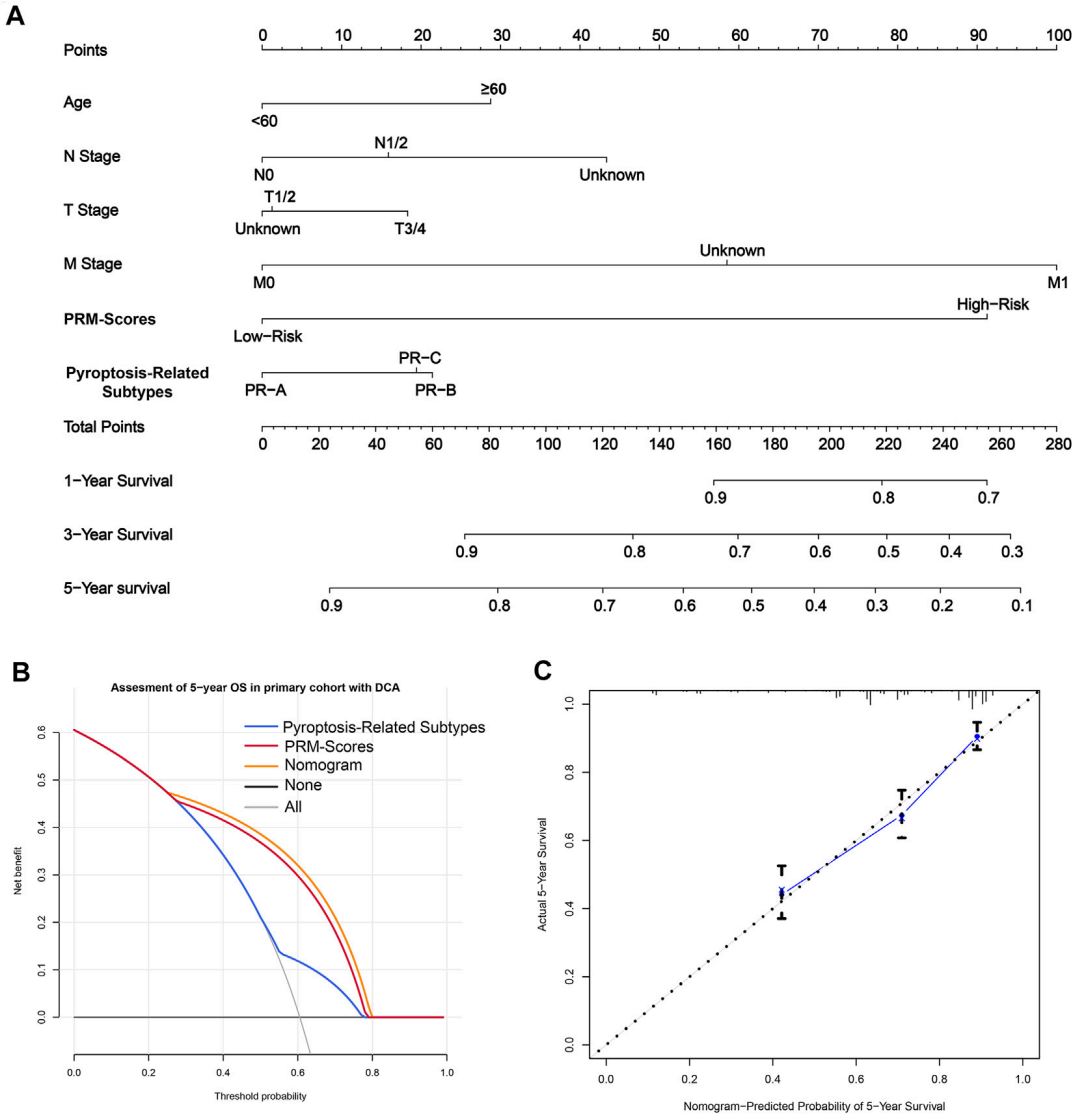
The clinical application value of the PRM-scores scoring model and pyroptosis-related (PR) clusters. **(A)** A nomogram was established for predicting 1, 3, and 5-years overall survival (OS) in colon cancer. To calculate probability of OS, first, determine the value for each factor by drawing a vertical line from that factor to the points scale. “Points” is a scoring scale for each factor, and “total points” is a scale for total score. Then sum up all of the individual values and draw a vertical line from the total points scale to the 1, 3, and 5-years OS probability lines to obtain OS estimates. **(B)** The decision curve analysis (DCA) of nomogram in inner-training set for 5 years OS. **(C)** Calibration curves for the probability of OS at 5 years. The nomogram cohort was divided into three equal groups for validation. The gray line represents the perfect match between the actual (*y*-axis) and nomogram-predicted (*x*-axis) survival probabilities. Black circles represent nomogram-predicted probabilities for each group, and X’s represent the bootstrap-corrected estimates. Error bars represent the 95% CIs of these estimates. A closer distance between two curves suggests higher accuracy.

Because stage-N, age, stage-M, PRM-scores, and PR subtypes were predictive for OS in multivariate analysis, these variables were further included in the nomogram, which was for predicting the 1, 3, and 5-years OS for CC patients (Figure 7A). The weighted total score, calculated from these factors, was applied to predict the 1, 3, and 5-years OS of CC patients.

Besides, the model showed good accuracy for predicting the OS, and internal validation was performed using the inner-training cohort with a C-index of 0.739. Furthermore, the decision curve analysis (DCA) results of the nomograms also confirmed their clinical applicability for predicting the OS, with superior performance compared with PRM-scores and PR subtypes (Figure 7B). Calibration curves for the probability of OS at 3 and 5 years indicated satisfactory consistency between actual observation and nomogram-predicted OS probabilities in CC cohort (Figure 7C, Supplementary Figure 4B).

## Discussion

Pyroptosis is a newly discovered type of programmed cell death induced by inflammasomes, leading to membrane rupture and the release of cell contents that trigger the inflammatory response. It has a dual function in tumor development, inhibiting tumor growth in liver cancer and having an ambiguous effect in breast cancer (Tan et al., 2021). Gasdermin family proteins are the executors of pyroptosis, which is regulated by multiple signaling factors and stromal cells in the TME. A comprehensive bioinformatics analysis of PR regulators is needed to evaluate the involved molecular signatures and signaling pathways, promising better results than those obtained when judging the prognosis using individual gasdermin proteins. Therefore, we evaluated the factors and molecular signatures related to pyroptosis to establish a classification and prognostic model, which provides potential signatures for CC therapy targeting pyroptosis.

In this study, we revealed three distinct pyroptotic tumor subtypes based on the expression of 27 PR regulators. These three subtypes had a significantly distinct prognosis, immune cell infiltration, and molecular characteristics. The PR-A subtype was characterized by a survival advantage, high immune-scores, and abundant immune cell infiltration, corresponding to an immunologically activated phenotype. The PR-B subtype corresponded to an intermediate phenotype. Finally, the PR-C type was characterized by a low immune response and high tumor purity, corresponding to an immune-excluded phenotype. According to the functional enrichment analysis, PR-C tumors exhibited low immune-scores and IL-17 production, T cell-mediated cytotoxicity, T-cell chemotaxis, and T-cell migration and differentiation, which were related to immune suppression, including the downregulation of NK cells, reduced B-cell proliferation, and subdued T-cell activation.

In order to provide a theoretical basis for the clinical treatment of CC, we established a reliable risk assessment tool based on three PR subtypes. The PS-score takes into account the heterogeneity of patients and links pyroptosis with the clinical prognosis. The PRM-scores were estimated based on the fractions of eight genes from the PR key module, and it featured both tumor promoter and suppressors, which were weighted differently. CTLA4, a member of the immunoglobulin superfamily, has been proved to act as an immunosuppressor that can convey the inhibitory signal to T cells in most tumors (Liu et al., 2021a; Sena et al., 2021). The treatment with immune checkpoint inhibitors (ICIs) against CTLA4 could reinvigorate the exhausted antitumor immunity (Wang et al., 2021a; Imazeki et al., 2021). Our results showed that CTLA4 expression is related to the tumor infiltration characteristics of multiple immune cell types. CCL11, a neutrophil-related chemokine, exerts a chemotactic effect on eosinophils by interacting with CXCR3 and CCR5 (Wang et al., 2021b), which was found to be a potential prognostic signature for TNM stage II CC patients (Liu et al., 2021b). NIN is essential for the construction of the centrosome and helps regulate cell migration and polarity (Goldspink et al., 2017). SNPs of NIN were found to be related with the morbidity of CRC (Grosch et al., 2013). The research on KIAA1671, CDH19, and TMEM154 mainly focused on their prognostic implications (Blons et al., 2002; Fernández-Madrid et al., 2004; Zhang et al., 2020). It was reported that CDH19 was related to the inflammatory response (Oparina et al., 2015). KIF7 is a member of the kinesin family that plays a significant role in cancer proliferation (Yao et al., 2019). TME cell infiltration data demonstrated that the PS-score holds an important value for immunotherapy. More activated immune cell infiltration in patients with a low PS-score predicted a better response to immunotherapy. Furthermore, we established an efficient and accurate nomogram to guide subsequent treatment for CC patients.

Finally, there are also some limitations that should be kept in mind when considering this research. Although we used multi-database searches to perform the verification from multiple angles, all of the database searches were retrospective and lacked complete clinical information. It is necessary to conduct prospective studies and perform subgroup validation. Furthermore, there is little current research on the role of pyroptosis in CC, and our research can only provide preliminary theoretical support for future experimental verification. The risk model developed in this study did not exhibit a better predictive value for the OS of CC patients, and the random survival forest algorithm exhibited overfitting and high variance. We plan to implement a more suitable machine learning method to improve the predictive ability.

In conclusion, we conducted a comprehensive and systematic bioinformatics analysis for PR regulators and demonstrated their relationship with the development of CC. This study also suggests the extensive effect of PR regulators on the tumor immune microenvironment based on the established PR CC subtypes. Moreover, we identified eight PR independent risk signatures, and we built the PRM-score for assessing the prognosis of CC patients. Our comprehensive evaluation of PR regulators improves our understanding of the TME and provides an important theoretical basis for prognosis and selection of therapeutic strategies.

## Data Availability

The datasets presented in this study can be found in online repositories. The names of the repository/repositories and accession number(s) can be found in the article/Supplementary Material.
