# Supplementary material for: Deciphering the Pyroptosis-Related Prognostic Signature and Immune Cell Infiltration Characteristics of Colon Cancer
Source: Front Genet. 2021 Oct 12;12:755384. doi: 10.3389/fgene.2021.755384 (PMC8546261; doi:10.3389/fgene.2021.755384)
Supplement: Supplementary file 2 [file DataSheet1.docx]

**
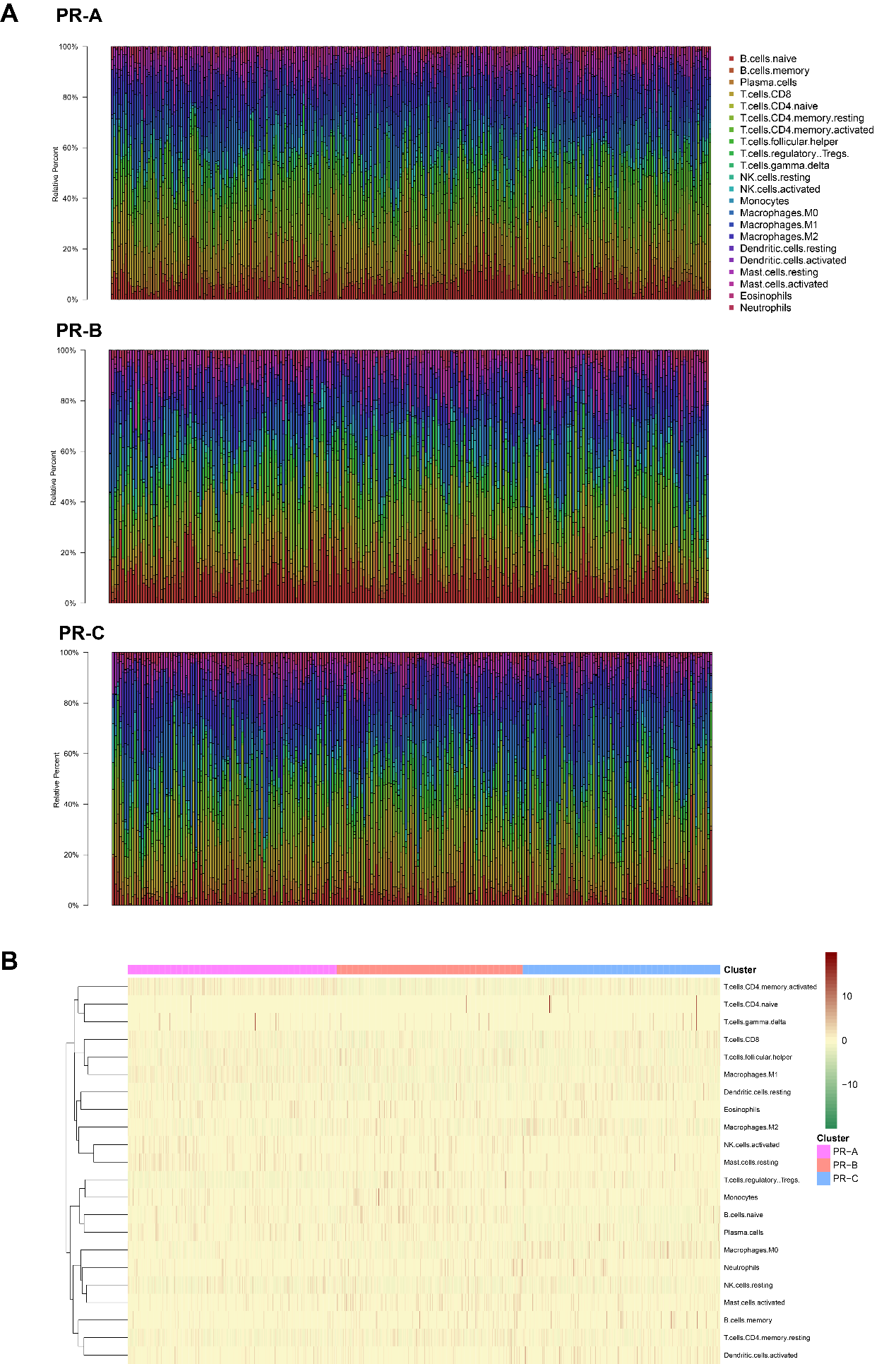
Supplementary Figures**

**Supplementary Figure 1.** Composition of immune cells in different pyroptosis-related group. (A) Barplot showing the fractions of 22 immune cells of colon cancer patients in three colon cancer databases. Column names of plot were sample ID. (B) Heatmap showing the level of immune cells by different pyroptosis-related group of colon cancer.


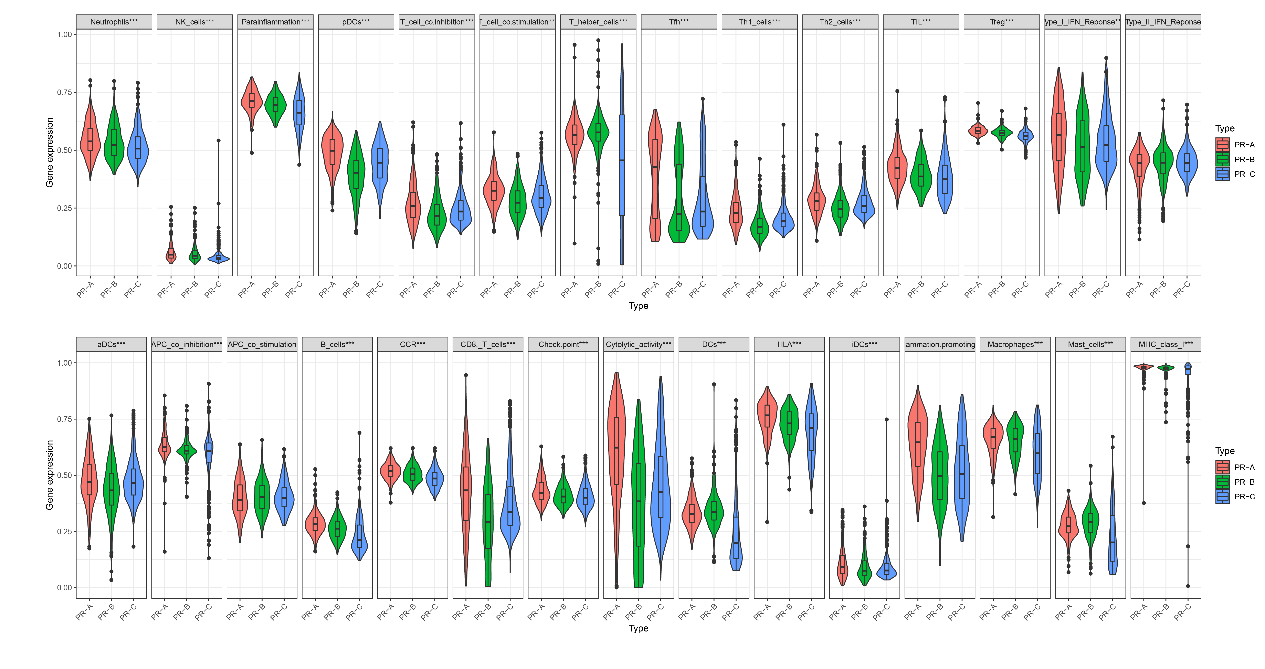


**Supplementary Figure 2.** Comparison of the immune cell infiltration levels between different pyroptosis-related group of colon cancer with ssGSEA. Mann–Whitney U test.

**
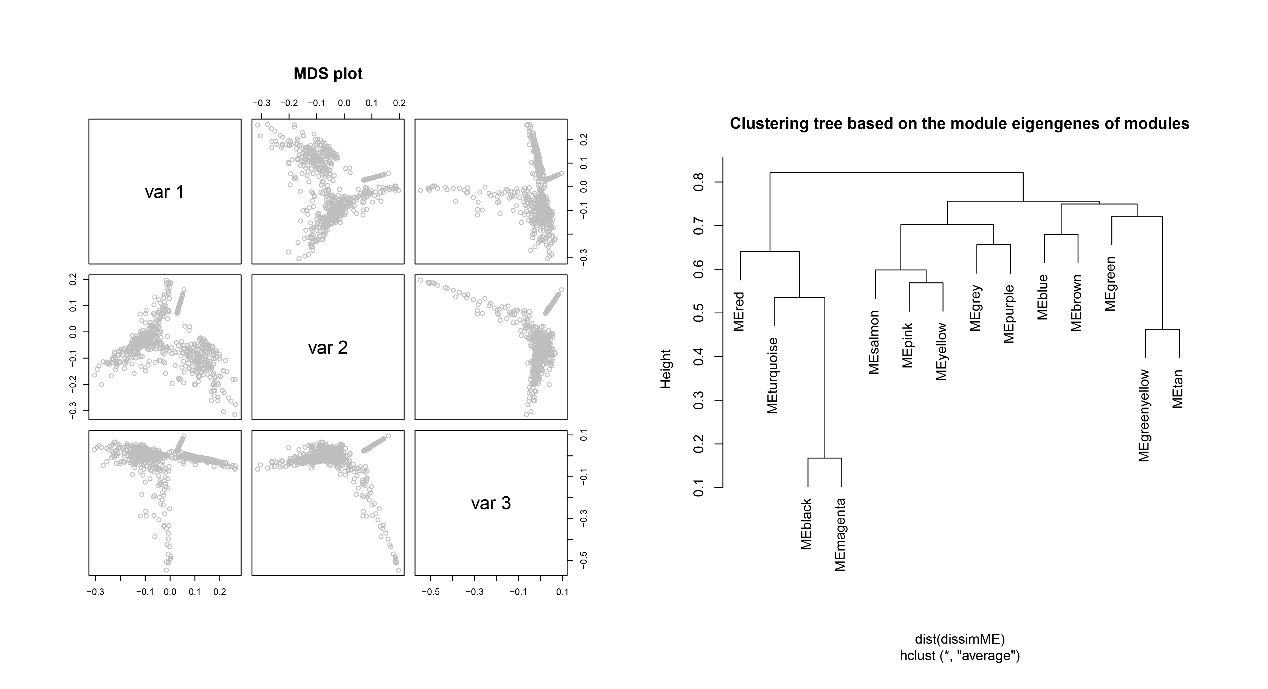
**

**Supplementary Figure 3.** WGCNA for colon cancer.

**
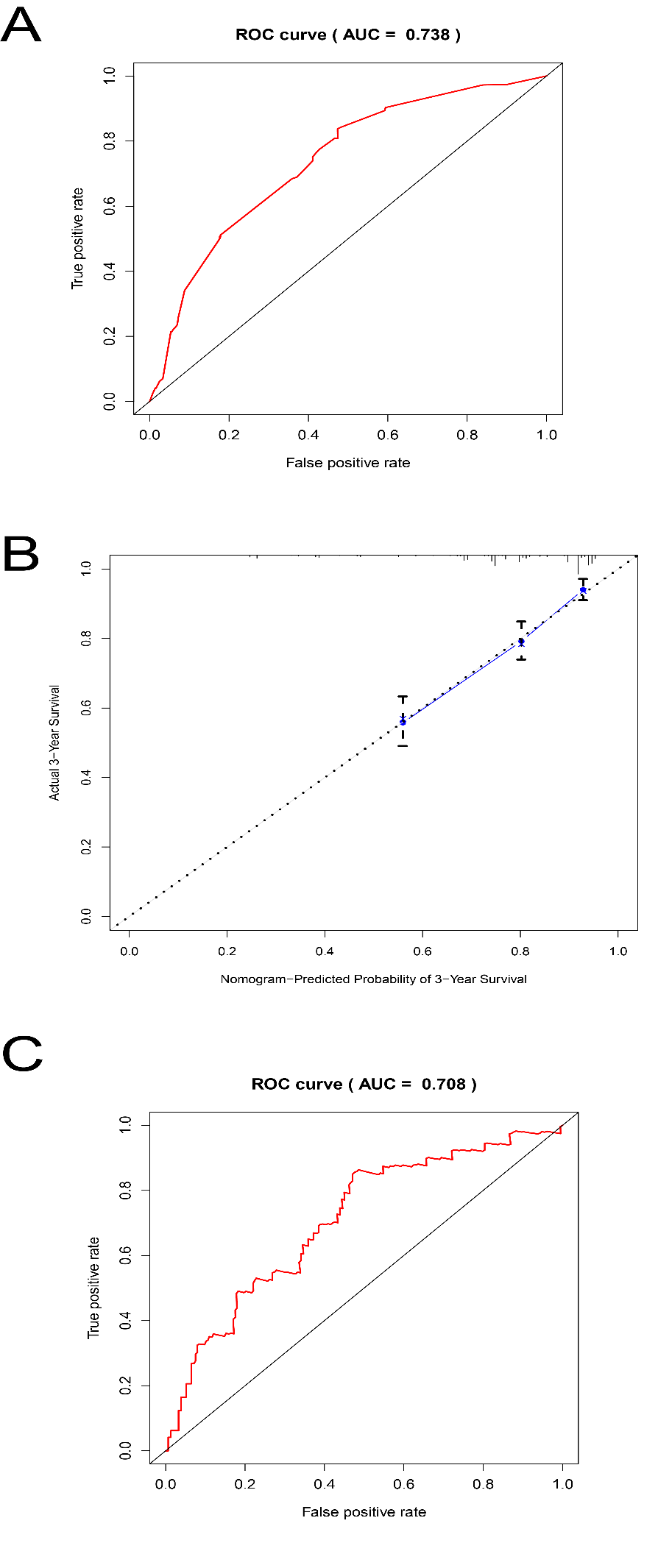
**

**Supplementary Figure 4.** (A-B) The prognostic value of the risk score showed by the time-dependent ROC curve for predicting the 3 years overall survival in training group; (C) The prognostic value of the risk score showed by the time-dependent ROC curve for predicting the 3 years overall survival in validation group.


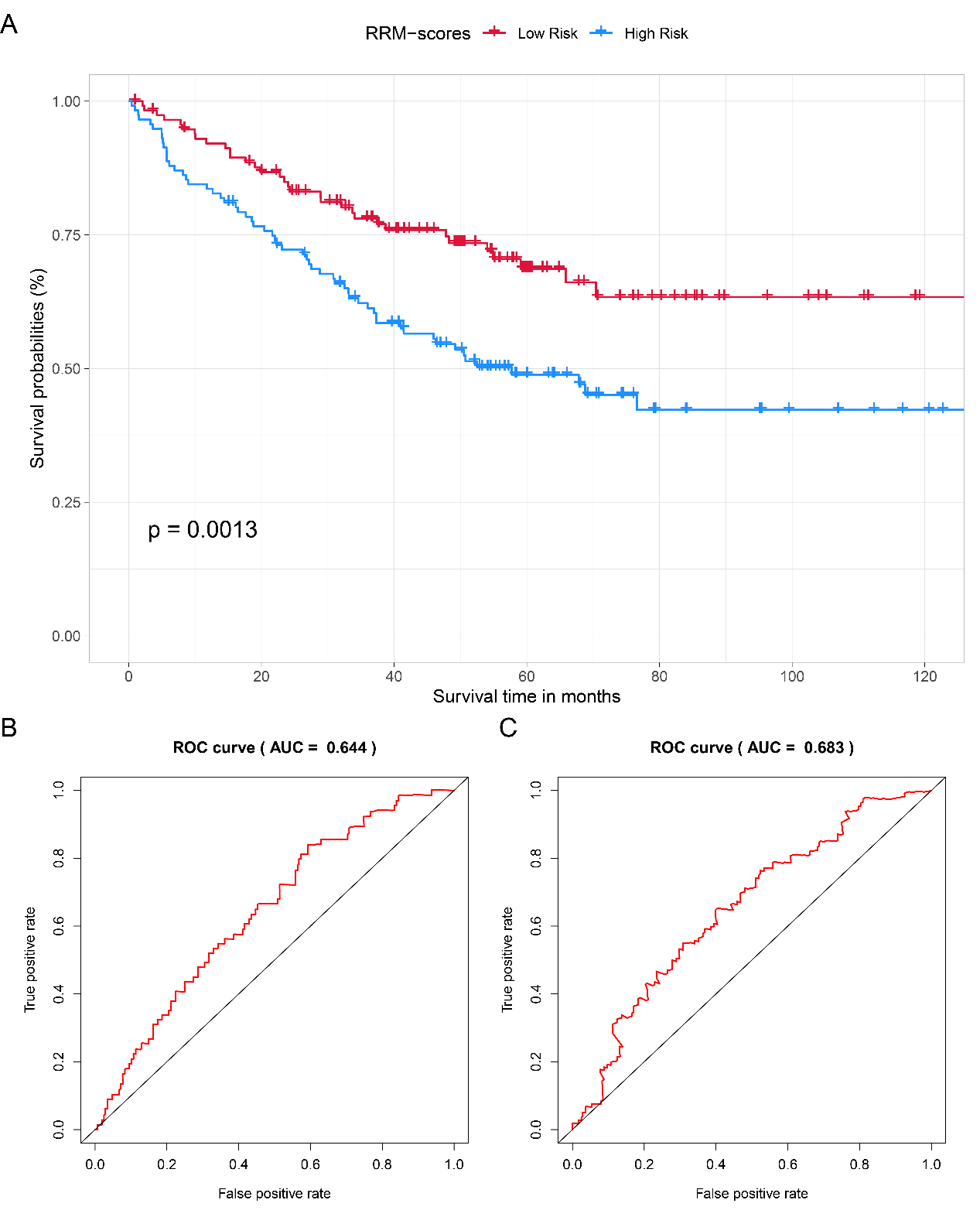


**Supplementary Figure 5.** (A)Kaplan–Meier curves for the OS of colon patients in the independent validation cohort (GSE17538) between the high- and low- PRM-scores groups (Log-rank test, p<0.01). (B) The prognostic value of the risk score showed by the time-dependent ROC curve for predicting the 3 years overall survival in the independent validation cohort (GSE17538). (C) The prognostic value of the risk score showed by the time-dependent ROC curve for predicting the 5 years overall survival in the independent validation cohort (GSE17538).


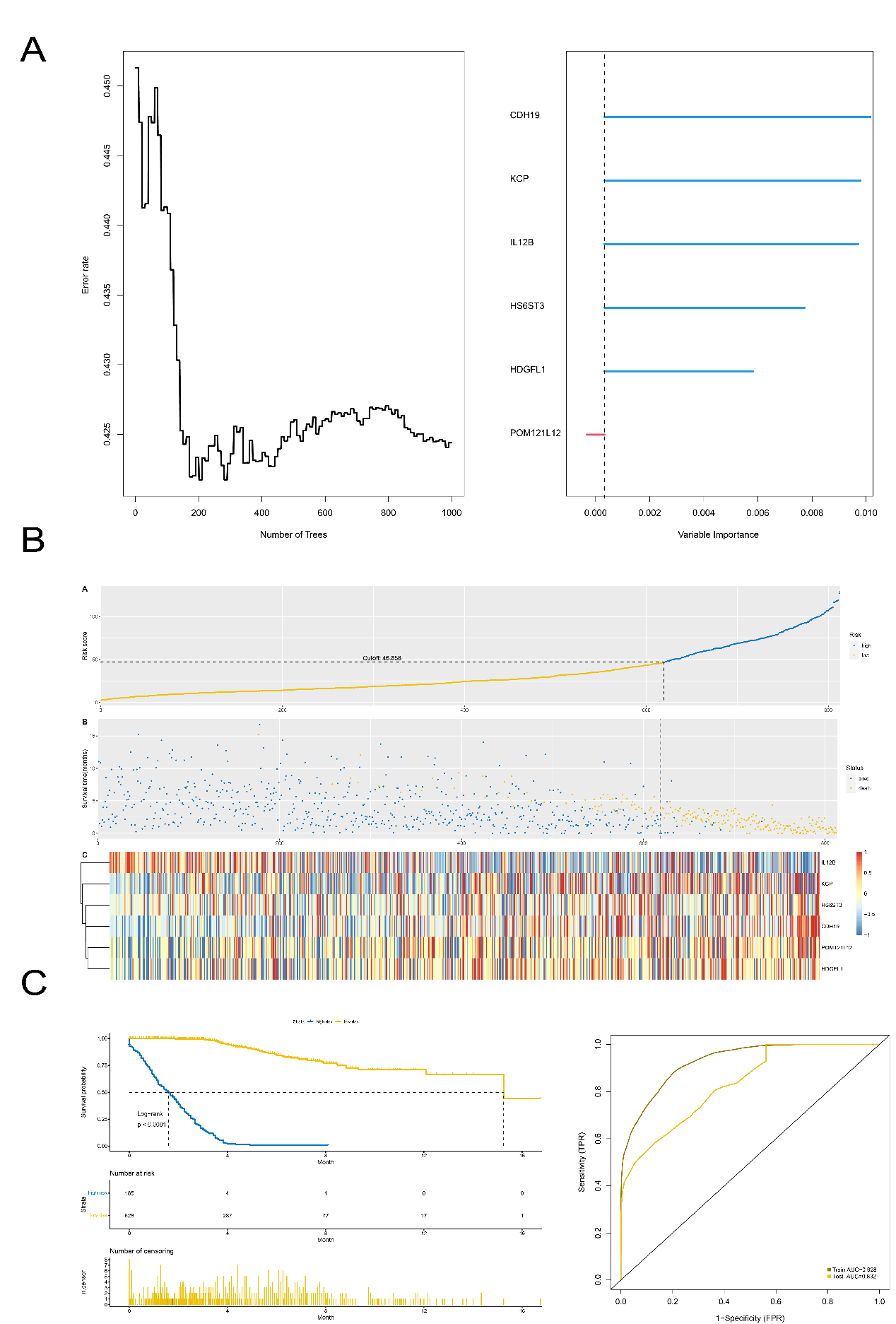


**Supplementary Figure 6.** (A) The prognosis model established based on the pyroptosis-related gens with “random Survival Forest”, and six genes were identified. (B) Risk score curve of the six-gene signature of colon cancer in training group; Heatmap showed the expression of six genes by risk score of colon cancer in training group. (C)The Kaplan–Meier test of the risk score for the overall survival of colon cancer between high-risk and low-risk patients in training group (log-rank test, p < 0.0001); The prognostic value of the risk score showed by the time-dependent ROC curve for predicting the 5 years overall survival in training and testing group

**
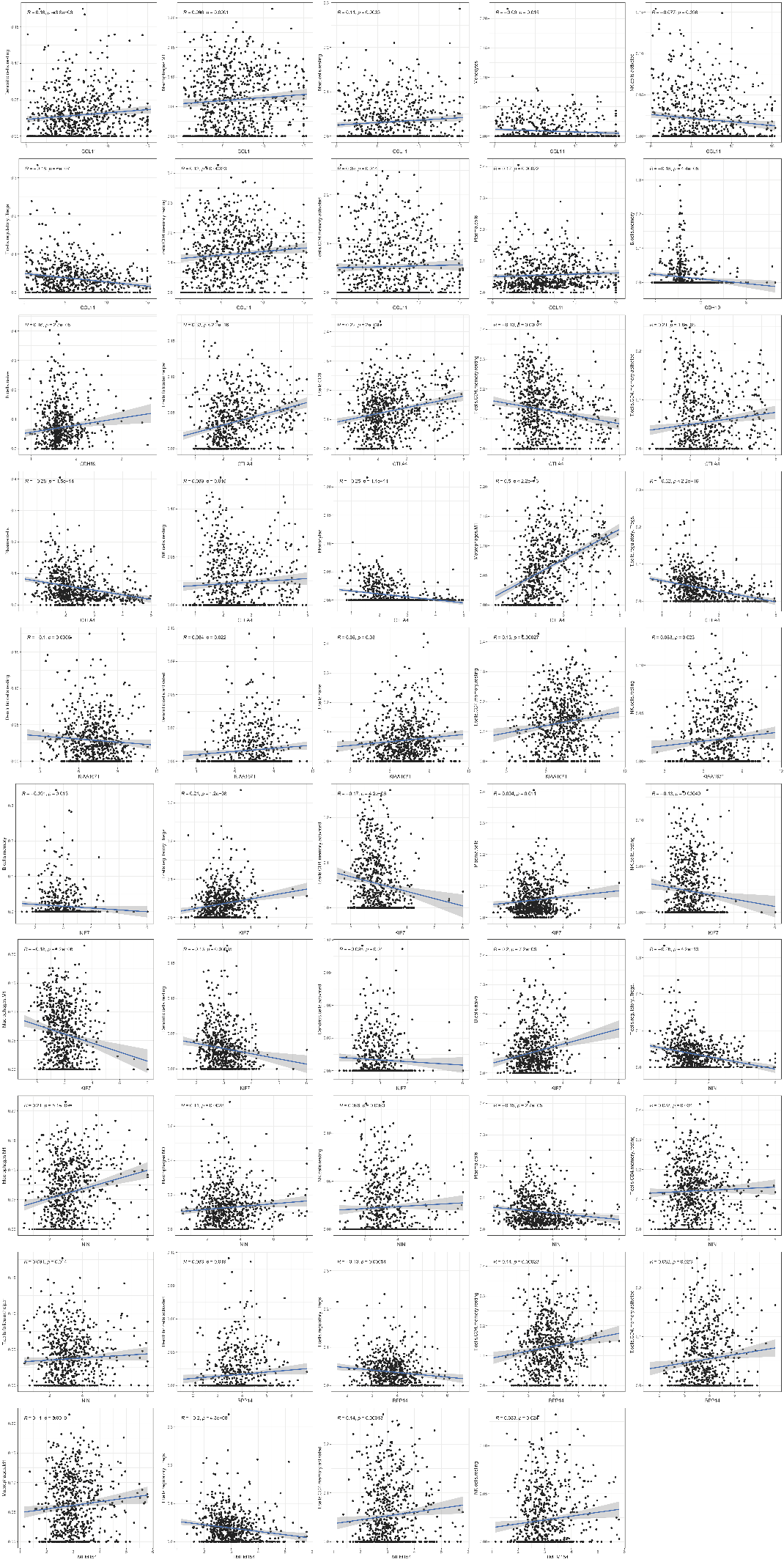
**

**Supplementary Figure 7.** The corrections between the eight signature genes and 22 types of immune cell infiltration profiles.
